# Styrene-Lauryl Acrylate Rubber Nanogels as a Plugging Agent for Oil-Based Drilling Fluids with the Function of Improving Emulsion Stability

**DOI:** 10.3390/gels9010023

**Published:** 2022-12-28

**Authors:** Hongyan Du, Kaihe Lv, Jinsheng Sun, Xianbin Huang, Haokun Shen

**Affiliations:** 1 Department of Petroleum Engineering, China University of Petroleum (East China), Qingdao 266580, China; 2 Key Laboratory of Unconventional Oil & Gas, Development Ministry of Education, Qingdao 266580, China

**Keywords:** oil-based drilling fluid, nanogels, plugging agent, emulsion stability

## Abstract

With the exploration and development of unconventional oil and gas, the use frequency of oil-based drilling fluid (ODF) is increasing gradually. During the use of ODFs, wellbore instability caused by invasion of drilling fluid into formation is a major challenge. To improve the plugging property of ODFs, nano-sized poly(styrene-lauryl acrylate) (PSL) rubber nanogels were synthesized using styrene and lauryl acrylate through soap-free emulsion polymerization method and were characterized using FTIR, NMR, SEM, TEM, particle size analysis and TGA. The results show that, due to good dispersion stability and oil-absorbing expansion ability, the PSL rubber nanogels have a wide range of adaptations for nano-scale pores to deposit a layer of dense filter cake on the surface of filter paper with various pore diameters, reducing the filtration of mineral oil and W/O emulsion significantly. Due to the unique wettability, the PSL rubber nanogels can be adsorbed stably at the oil–water interface and form a dense granular film to prevent droplets coalescing, which improves the emulsification stability of W/O emulsion. Furthermore, the PSL rubber nanogels are soap-free and compatible with ODFs without foaming problems. The PSL rubber nanogels can increase the hole-cleaning performance of ODFs by raising viscosity and yield point. The PSL rubber nanogels outperformed hydrophobic modified nano silica and polystyrene nanospheres in plugging and filtration reduction. Therefore, the PSL rubber nanogels are expected to be used as a new plugging agent in oil-based drilling fluid. This research provide important insights for the use of organic nanogels in ODFs and the optimization of plugging conditions.

## 1. Introduction

In the past decade, unconventional oil and gas resources, represented by shale gas, have attracted more attention [1,2,3]. However, in the process of shale gas drilling, wellbore instability in shale gas formation is one of the major challenges [4]. According to literatures, about 90% of wellbore instability accidents occur in the drilling process of shale formation [5,6,7]. There are a large number of pores and fractures in shale formation [8]. The water in the drilling fluid enters the formation through these channels and contacts with clay minerals in shale will lead to serious hydration and expansion of clay minerals, causing complex downhole accidents, such as sticking of drilling tools and wellbore collapse [5,6,9,10]. Moreover, as one of the drilling techniques to improve shale gas production, horizontal wells also increase the risk of wellbore instability [11]. Therefore, improving wellbore stability of shale gas formation has become one of the research focuses of shale gas exploration and development.

As a drilling fluid with mineral oil as the continuous phase, oil-based drilling fluid (ODF) is widely used in shale oil and gas drilling due to its excellent inhibition, temperature resistance, lubrication and pollution resistance [12]. However, in the process of drilling with ODF, wellbore instability still occurs frequently. The research shows that, when the oil phase in ODF enters the formation through the pores and fractures in the shale, the pore pressure in the shale pores will increase significantly, which will reduce the supporting force of drilling fluid on the wellbore and cause mechanical instability and failure of the wellbore [13]. In addition, the invasion of the oil phase will reduce the friction between fractures and joints and weaken the binding force of wellbore, leading to wellbore instability [14,15,16]. Therefore, developing high-performance ODF plugging agent to reduce the invasion of drilling fluid to the formation and to improve the plugging performance of ODF is one of the key methods to solve the wellbore instability [17].

Generally, commercial plugging agents for ODFs mainly include silica and its modified products [18], ultra-fine calcium carbonate [19], resin [20], modified graphene [21] and asphalt [22]. However, due to the large particle size, poor dispersion and strong rigidity, inorganic plugging agent cannot effectively plug the nano pores in shale formation. When using asphalt materials, the softening point of asphalt is required to adapt to the formation temperature [22]. However, in actual operation, it is difficult to determine the softening point, and asphalt resin will affect the rheology of drilling fluid, which is not conducive to improving the penetration rate.

In recent years, with the application of nano materials in drilling fluid, nano organic plugging agent has been proved to play an important role in reducing rock permeability, reducing the filtration of drilling fluid to the formation and improving the wellbore stability [18,23,24,25]. Nano organic plugging agent is considered the next-generation plugging material because of its good thermal stability, good dispersion stability, uniform particle distribution, controllable particle size, and easy access to shale micro fractures to plug nano pores [26,27,28]. It has been extensively studied for its application in water-based drilling fluids [24,29,30,31,32]. However, there is little research on the application of nano organic plugging agent in ODFs. Wang et al. [28] used styrene and methyl methacrylate through emulsion polymerization to synthesize nano particles as the plugging agent for ODF and showed good plugging effect. Li et al. [33,34] synthesized a styrene butadiene resin/nano-SiO_2_ composite as a nano-plugging agent for ODFs, which can reduce the filter loss and pressure transmission. Geng et al. [14] synthesized a surface-modified nano-scale polystyrene material as a plugging agent for OBFs, which can improve the contact angle of the mineral oil on the rock surfaces, reduce the amount of imbibition of the oil into the rock, and maintain the wellbore stability. Xie et al. [35] prepared an oil-soluble polyamine as a nano plugging agent, which can decrease the permeability of artificial cores and slightly increase the emulsion stability of OBFs.

However, the synthesis method of nano organic plugging agent mainly depends on emulsion polymerization. In order to ensure the stability of the reaction system, sufficient emulsifiers are usually added. After the reaction, these residual emulsifiers and plugging agent products added to the drilling fluid and will cause serious foaming of the drilling fluid, resulting in a reduction in the density of the drilling fluid. The soap-free lotion polymerization of conventional lotion polymerization can effectively eliminate the shortcomings of emulsifiers. Moreover, the existing nano organic plugging agents have weak deformability and adaptability to pores of different scales [31].

**Scheme 1:**
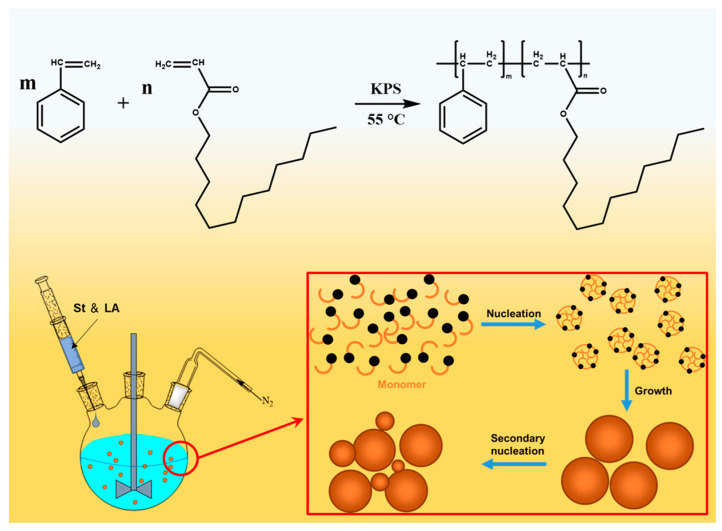
The chemical reaction formula to synthesize PSL.

This research aims to develop an organic plugging agent that can maintain stable dispersion in mineral oil and effectively plug the nano pores of shale to improve the wellbore stability. In this work, we prepared poly(styrene-lauryl acrylate) (PSL) rubber nanogels as the plugging agent of oil-based drilling fluid through soap-free emulsion polymerization, as shown in Scheme 1. Its chemical structure, morphology, thermal performance, surface wettability, colloidal stability, swelling behavior and emulsion stability were characterized. We were surprised to find that the polymer nanospheres can improve the stability of invert emulsion at high temperature. In addition, a series of experiments were conducted to evaluate the plugging performance and the compatibility with ODF. This study can provide important insights for the application of organic nanoparticles in oil-based drilling fluids and the optimization of plugging conditions.

## 2. Results and Discussion

### 2.1. Characterization of PSL Rubber Nanogels

**Figure 1:**
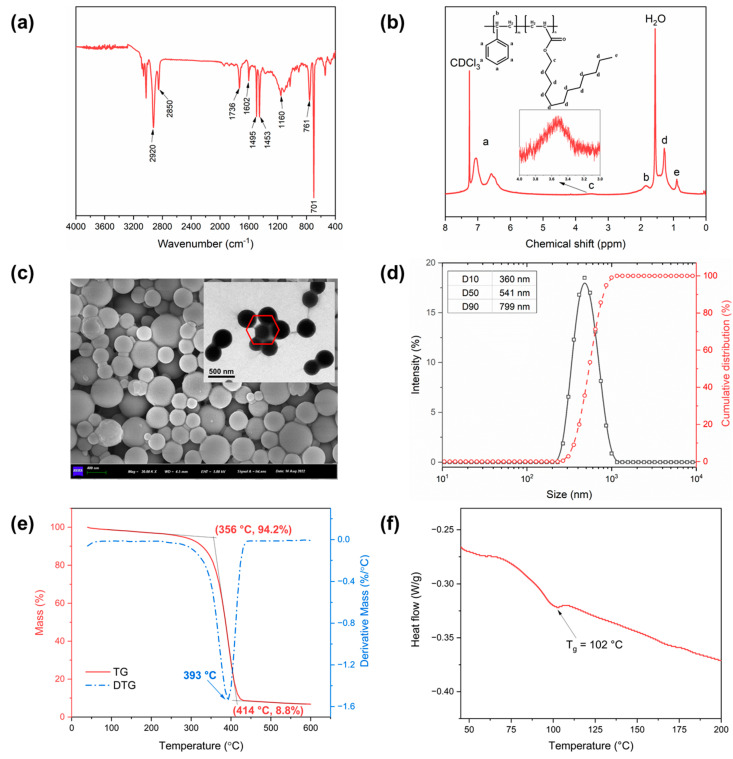
FTIR spectrum (**a**), ^1^H NMR curves (**b**), micromorphology images (**c**), particle size distribution curve (**d**), TG analysis (**e**), DSC curve (**f**) of PSL rubber nanogels.

FT-IR and ^1^H NMR spectroscopy were performed to clarify the structure of PSL rubber nanogels. The FT-IR spectra of PSL rubber nanogels was presented in Figure 1a. The absorption peaks at 1453 cm^−1^, 1495 cm^−1^ and 1602 cm^−1^ were attributed to C=C stretching vibration peak in benzene ring. The absorption peaks at 701 cm^−1^ and 761 cm^−1^ were attributed to the out-of-plane bending vibrations of hydrogen in the benzene ring, which are the characteristic bands of monosubstituted benzene. The peak at wavelengths of 1736 cm^−1^ was caused by the C=O in the ester group. The absorption peaks at 2920 cm^−1^ and 2850 cm^−1^ were attributed to asymmetric CH_3_ stretching and symmetrical CH_2_ stretching. In addition, the ^1^H NMR spectroscopy of PSL rubber nanogels was shown in Figure 1b. The prominent peak at 7.26 ppm is from the solvent (CDCl_3_), and the sharp peak at about 1.5 ppm is due to water. The multiple peaks (peak a) from 6.5 to 7.2 ppm are the chemical shifts of protons in the benzene ring. The peak at b from 3.2 to 3.5 ppm should be attributed to the C-H in the main chain of the copolymer. The peaks at (c) and (d) are associated with the protons of the C-H from the lauryl acrylate. In particular, the proton peaks at (c) have a higher chemical shift due to the effect of ester groups. Peak at (e) are associated with methyl in lauryl acrylate. Both the FT-IR and ^1^H NMR spectroscopy indicated that the resulting product was the target product.

In order to observe the morphology and particle size of PSL rubber nanogels, SEM, TEM, and the evaluation of particle size distribution were carried out. The SEM image and TEM image (Figure 1c) clearly show that the nanogels were spherical in shape and have a smooth surface. However, a certain level of aggregate phenomenon can be observed. In addition, some of the particles also had a hexagonal shape, indicating that the nanogels were flexible and deformed. A wide particle size distribution (200–1000 nm) can be observed from Figure 1d, and the median particle size (D_50_) of nanogels was 541 nm, which can plug holes and fractures of different sizes.

Since drilling fluid is usually used in a high-temperature environment, excellent thermal stability of drilling fluid additives is an important trait. To demonstrate the thermal property of PSL rubber nanogels, thermogravimetric analysis (TGA) and differential scanning calorimetry (DSC) were carried out in a N_2_ atmosphere. As shown in Figure 1e, the weight reduction of nanogels can be divided into three stages. The first stage of weight loss occurred between room temperature and 356 °C, and the slope of the TGA curve remained almost unchanged. The weight loss rate before 356 °C is only 5.8%, which is due to the solvent volatilization in the nanogels. In the second stage (356–414 °C), the weight of PSL rubber nanogels decreased sharply and the DTG curve occurred a peak at 393 °C, indicating that the nanogels began to degrade thermally. When the temperature reaches 414 °C, the residual mass is only 8.8%, indicating that the purity of PSL rubber nanogels is high. At a temperature of > 414 °C (the third stage), PSL rubber nanogels were converted to carbon, and the TGA curve becomes stable. TGA results show that PSL rubber nanogels have good thermal stability. This is due to the strong rigidity of the benzene ring in the nanogels, which limits the molecular thermal movement of the polymer molecular chain in the high-temperature environment. In addition, the glass transition temperature (T_g_) was characterized by DSC (Figure 1f). According to the DSC curve, the softening point of PSL rubber nanogels is 102 °C. The DSC curve of the sample showed a single glass transition, indicating that the completely mixed random copolymer formed a single phase. Generally, the temperature of shale formation in Sichuan Basin is around 100–120 °C, which is higher than the softening point of PSL rubber nanogels. During the formation process, the nanogels can be in the rubber state. Driven by the positive pressure difference between the drilling fluid and the formation, they can be pressed into pores or fractures to form tight plugs.

### 2.2. Dispersion Stability of PSL Rubber Nanogels

**Figure 2:**
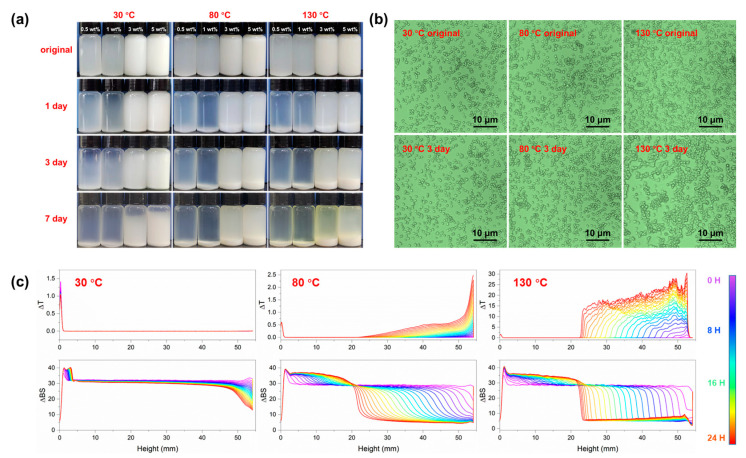
(**a**) Photographs of mineral oil dispersions with different concentration of PSL rubber nanogels at various temperature captured on different storage days (specified at the left, up to 7 days). (**b**) Optical microscope images of mineral oil dispersions with 5 wt% PSL rubber nanogels at various temperature for 3 days. Before observation, the dispersion was diluted 50 times using mineral oil. (**c**) NIR transmittance (∆T) and backscattering (∆BS) results for the mineral oil dispersions with 5 wt% PSL rubber nanogels at various temperatures obtained every 30 min for 12 h (the color of each curve represents its measurement time, as denoted in the spectral column on the right). The x-axis indicates the sample height from 0 (bottom) to 55 mm (top).

Photographic images of nonaqueous suspension added with PSL rubber nanogels of different concentrations placed at different temperatures are presented in Figure 2a. All the original samples were uniformly ivory, indicating the initially uniform dispersion of the PSL rubber nanogels in mineral oil. For the dispersions at 30 °C, a fuzzy oil phase appeared near the top of the sample within 3 days, and around one fifth of the particles precipitated within 7 days. For the dispersions at higher temperature, more sediment has settled at the bottom of the bottle, implying a faster settling speed. However, the upper dispersion kept high turbidity throughout the test period. This may be due to the existence of a considerable number of extremely small nanogels in the dispersion, and the Brownian motion of these small nanospheres is intensified due to the increase in temperature. Meanwhile, to study the dispersion state of the nanospheres in mineral oil, optical microscopy was used. The results are shown in Figure 2b. It can be seen that the dispersion state of the original dispersion under different temperatures is roughly the same, and the nanogels are separated from each other without large-scale aggregation, which indicates that the PSL rubber nanogels have good dispersion in white oil. After three days of storage, the sample at 30 °C did not change significantly. However, for the sample at 130 °C, the number of particles increased significantly and agglomerated to a certain extent, indicating that the colloidal stability of the nanogels was weakened. This trend is consistent with Figure 2a. In addition, an 880 nm near-infrared (NIR) light was periodically emitted onto an independent sample having the highest surfactant concentration throughout the sample height, and the intensities of transmitted and backscattered (45°) light were collected over 12 h, as shown in Figure 2c. Firstly, for the sample at 30 °C, no increase in the transmitted light along with the sample height was observed, except in the bottom layer, during the same measurement duration, which indicates low sedimentation. In the sample at 130 °C, while the transmitted light increased rapidly with time due to particle sedimentation, the backscattered light in the middle of the sample also gradually increased, indicating that the particles were aggregating and that the Mie scattering of large aggregates was occurring [36]. The increase in the backscattered light after 8 h is attributed to the reflection of the first wall-transmitted light by the second wall owing to the clarification of the sample, which was consistent with the visible observation after 8 h. There was a minor increase in the backscattered light in the middle of the sample, which can be attributed to the increased Rayleigh scattering due to the formation of small aggregates, in contrast to the other two samples, wherein the Mie scattering of large aggregates was dominant. All in all, the dispersions at 30 °C exhibited the highest dispersion stability, and the dispersion stability declined with the increase in temperature.

### 2.3. Swelling Behavior of PSL Rubber Nanogels

**Figure 3:**
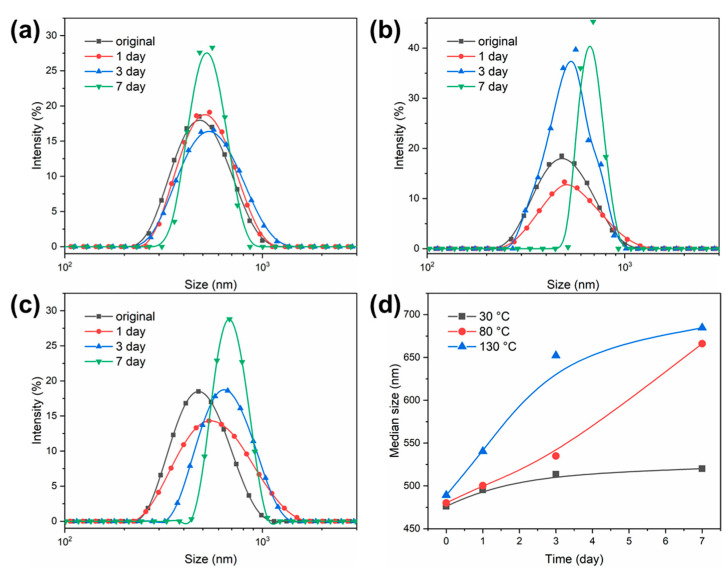
Swelling capability of the PSL rubber nanogels at different temperature for 7 days. (**a**) 30 °C, (**b**) 80 °C, (**c**) 130 °C. (**d**) The median size (D_50_) of the PSL rubber nanogels at different temperature for 7 days.

The swelling behaviors of the PSL rubber nanogels was studied, and their particle size distribution curves after absorbing mineral oil are shown in Figure 3. As shown in Figure 3a, the particle size of 30 °C increased slightly as time passed and finally reached swelling equilibrium after 7 days, when the median size increased from 476 nm to 520 nm. Furthermore, when the ambient temperature increased to 80 °C (Figure 3b), this swelling behavior is more obvious, and the median size increased from 489 nm to 684 nm after 7 days. In fact, due to the presence of a benzene ring and long-chain alkyl, mineral oil can penetrate into the PSL rubber nanogels. Before the polymer molecular chain diffuses into the solvent, the solvent molecules have diffused into the polymer molecules to cause its swelling. In addition, this infiltration is positively correlated with temperature. However, due to the use of crosslinking agent, when the polymer nanogels swell to a certain extent, the solvent absorption does not increase, and the polymer volume does not increase, reaching the swelling equilibrium [37]. It can be imagined that this swelling behavior is beneficial to improving the plugging effect when nanogels enter the pores.

### 2.4. Enhancement of Emulsion Stability

**Figure 4:**
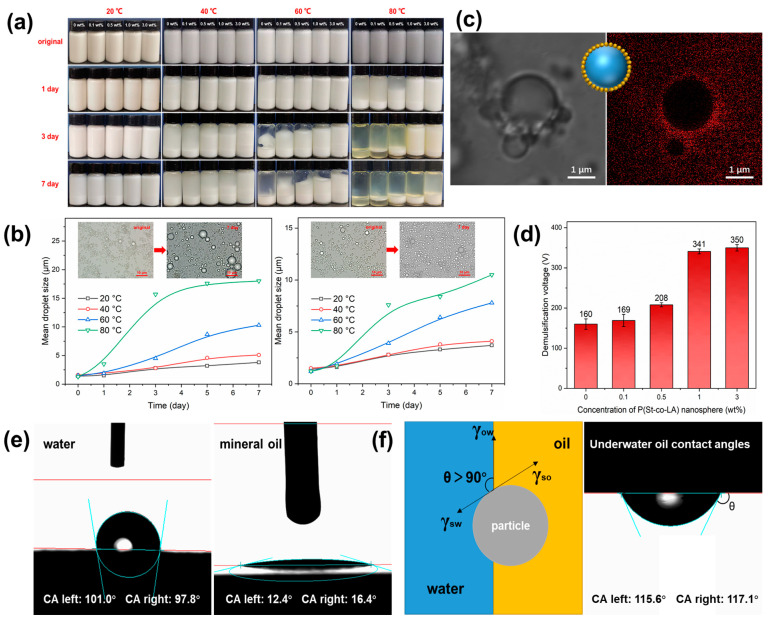
(**a**) Photographs of W/O emulsion with various PSL rubber nanogels concentrations (marked at the top) and (**b**) mean droplets size of W/O emulsion without PSL rubber nanogels (left) and with 3 wt% PSL rubber nanogels (right) captured on different storage days (marked at the left, up to 7 days) at different temperatures. (**c**) CLSM image of 3 wt% PSL rubber nanogels stabilized W/O emulsions. The red parts are PSL rubber nanogels stained by Nile Red. (**d**) Demulsification voltage of W/O emulsion with various PSL rubber nanogels concentrations. (**e**) Contact angle of water (left) and of mineral oil (middle) on the PSL rubber nanogels film in air and underwater oil contact angles of mineral oil (right) on the PSL rubber nanogels film. (**f**) Schematic diagram of the mechanism of PSL rubber nanogels improving the stability of emulsion.

Photographic images of the W/O emulsion added with PSL rubber nanogels of different concentrations placed at different temperatures are presented in Figure 4a. All the samples at the storage time (t) of 0 were uniformly ivory with a high turbidity, indicating the successful preparation of the emulsion and the successful dispersion of PSL rubber nanogels in the emulsion. However, the sedimentation behaviors of emulsions at different temperatures were different. For the emulsion placed at 20 °C without nanogels, the sample was slightly separated after 7 days. With the increase in ambient temperature, the separation became obvious. A clear oil phase could be observed at the top of the emulsion at 60 °C. When temperature increased to 80 °C, the emulsion was completely demulsified. The optical microscope study of emulsion droplets showed the same trend, the mean droplets diameter increasing from less than 2 μm to 3.8 μm (20 °C), 5.1 μm (40 °C), 10.3 μm (60 °C) and 18 μm (80 °C), respectively, after seven days (Figure 4b). It is due to the emulsion is a thermodynamically unstable system. The Brownian movement of the droplets in a high-temperature environment is intensified, and the probability of collision between the droplets is increased, which could lead to the coalescence of droplets in emulsion. However, when the concentration of nanogels increased from 0.1 wt% to 3.0 wt%, the stability of the emulsion was significantly improved. Especially for the emulsion containing 3% nanogels, the stable emulsion was still maintained after 3 days at 80 °C, and the mean particle size of droplets only increased to 7.6 μm.

In addition, in order to investigate further the contribution of PSL rubber nanogels to the stability of the emulsion, we conducted electrical stability (ES) test on the emulsion with different contents of nanogels. ES is determined by applying a voltage-ramped, sinusoidal electrical signal across a pair of parallel, flat-plate electrodes immersed in the emulsion. The resulting current remains low until a threshold voltage is reached, whereupon the current rises very rapidly. This threshold voltage is referred to as the ES of the oil-based drilling fluid and is defined as the demulsification voltage. As shown in Figure 4d, with the increase in the addition of PSL rubber nanogels in the emulsion, the demulsification voltage of emulsion gradually increases, which indicates that the electrical stability of the emulsion is improved. All these results indicated that PSL rubber nanogels can improve emulsion stability.

As we all know, the adsorption of some solid particles at the oil–water interface will directly affect the emulsion stability. This is due to the adsorption of particles at the oil–water interface, which forms a stable interface film to prevent the droplets from coalescing, so that the emulsion could maintain long-term stability [38]. To gain insight into the stability of a PSL rubber nanogels-stabilized emulsion, the emulsion was investigated with confocal laser scanning microscopy. The presence of red bright points surrounding each droplet in Figure 4c implies that the nanogels adsorbed at the oil–water interface and formed a densely packed particle layer on the droplets’ interface. Furthermore, the nanogels at the interface provided a steric barrier to prevent the coalescence of droplets. The adsorption behavior of PSL rubber nanogels at the oil–water interface can be attributed to the wettability of the nanogels. In air, the contact angle of deionized water and mineral oil on the surface of the nanogels was 101° and 12.4°, respectively, which revealed that the nanogels were lipophilic (Figure 4e). The lipophilicity determined that PSL rubber nanogels could be dispersed in mineral oil, which can be observed in Figure 2a. However, completely hydrophilic or hydrophobic particles have no interfacial activity. Only partially wetted particles have strong interfacial adsorption energy. The interfacial adsorption energy (∆*_ads_*G) can be calculated via

$$\Delta_{ads}G=\pi r^{2}\gamma_{OW}{\left(1+\cos\theta \right)}^{2}$$

where *r* is the particle radius, γ*_OW_* is the interfacial tension, and θ is the three-phase contact angle. From the adsorption energy calculation formula, the closer the θ is to 90°, the higher the desorption energy and the more stable the emulsion is. In addition, due to the difference in wettability between oil and water, most of the particles tend to be in the phase with better wettability, resulting in the interface bending to the phase with poor wettability, thus forming emulsion droplets. Most studies have shown that, when the θ of the particles at the oil–water interface is slightly greater than 90°, most of the particle surfaces will be in the oil phase, which makes the formation of water-in-oil (W/O) emulsions more likely (Figure 4f). The θ of the deionized water on PSL rubber nanogels surface was 117.1°, which conform to the adsorption energy theory [39].

### 2.5. Plugging Performance of PSL

The effect of PSL rubber nanogels on the plugging of microporous membranes with different pore sizes in two fluids (i.e., mineral oil and inverse emulsion) was evaluated via filtration loss experiments. The size of the actual shale formation pore throats is nanometer-scale, but the size of the filter paper for drilling fluid is micron-scale. In order to simulate the interaction between nanopores of the shale formation and the plugging agent, the PTFE microporous membranes were used to evaluate the plugging performance.

**Figure 5:**
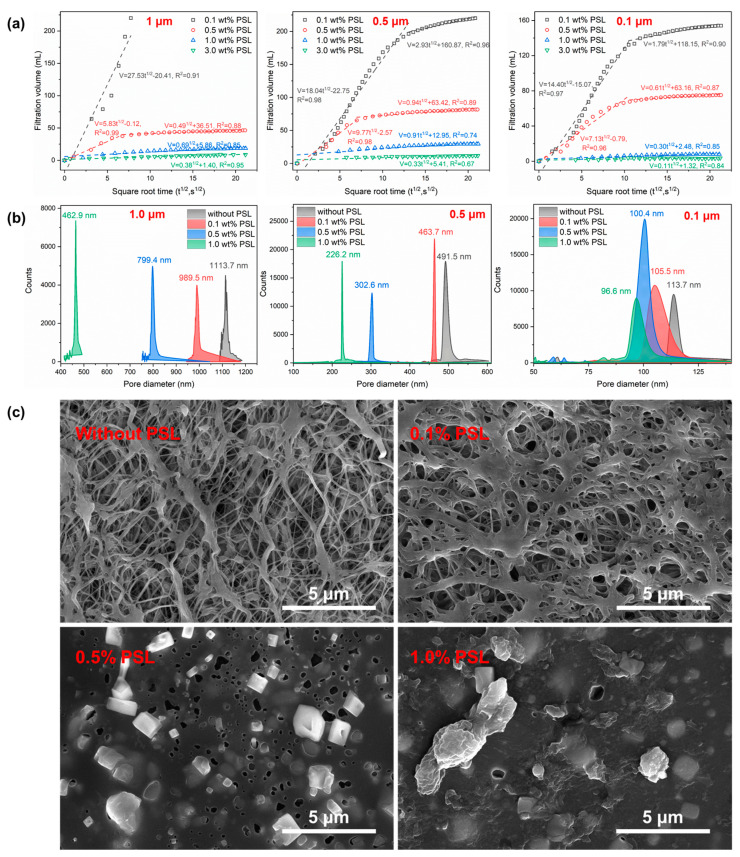
(**a**) The static filtration volume of mineral oil with different concentrations of PSL rubber nanogels as a function of the square root of time, (**b**) the pore diameter distribution of the different polytetrafluoroethylene membrane after filtration test, (**c**) SEM images of the polytetrafluoroethylene membrane (1.0 μm) after the filtration test. The magnification is 13,000 times.

Figure 5a present the curves of the filter loss of the PSL mineral oil dispersion with the square root of time. The experimental results shown that mineral oil with 0.1% PSL rubber nanogels quickly passed through the microporous membranes with pore sizes of 1 μm and was completely filtered through within 60 s. However, after adding 0.5% PSL, the filtration volume of the PSL mineral oil dispersion decreased significantly, and the filtration volume was 50 mL after 450 s. Moreover, the filtration volume of the mineral oil dispersion decrease with the further increase in the PSL rubber nanogels. For mineral oil dispersion with 3 wt% PSL rubber nanogels, the filtration volume through the 1.0 μm, 0.5 μm and 0.1μm pore size filters were 10.9 mL, 9.6 mL and 3.1 mL, respectively.

The filtration loss experiments uses static filtration. The characteristic of static filtration is that the drilling fluid is static, and the thickness of filter cake as one of the percolation media (another media is the filter membrane) is variable, which thickens with the extension of percolation time. Generally, the permeability of filter cake is far less than the permeability of filter membrane. If the ratio of the thickness of filter cake to the diameter of filter membrane is small enough, it can be assumed that the percolation is linear, which can avoid the use of complex percolation mode and is expressed by Darcy’s law. According to Darcy’s law, the filtration rate $dV_{f}/dt$ is linear to the permeability of filter cake K via

(1) $$\frac{dV_{f}}{dt}=\frac{KAp}{h\mu }$$

where A is the filtration area, p is the filtration pressure, h is the thickness of filtrate cake, μ is the filtrate viscosity, $V_{f}$ is filtration volume, and t is filtration time.

If a certain volume ($V_{m}$) of drilling fluid is filtrated completely, the following material balance will be achieved:

(2) $$V_{m}=hA+V_{f}$$

(3) $$V_{s}=hAC_{c}$$

where $V_{s}$ is the volume of solid phase in filter cake, and $C_{c}$ is the volume fraction of solid in filter cake.

According to Equations (5) and (6), the following equation can be obtained:

(4) $$C_{m}=\frac{V_{s}}{V_{m}}=\frac{hAC_{c}}{hA+V_{f}}$$

(5) $$h=\frac{V_{f}}{A\frac{C_{c}}{C_{m}}-1}$$

Thus,

(6) $$\frac{dV_{f}}{dt}=\frac{KA^{2}p\left(\frac{C_{c}}{C_{m}}-1\right)}{V_{f}\mu }$$

After integration, the following equation can be obtained:

(7) $$\frac{V_{f}{}^{2}}{2}=\frac{KA^{2}p\left(\frac{C_{c}}{C_{m}}-1\right)t}{\mu }$$

(8) $$V_{f}=A\sqrt{\frac{2Kp\left(\frac{C_{c}}{C_{m}}-1\right)}{\mu }}\sqrt{t}$$

According to Equation (11), the filtration volume can reflect the compactness of the filtrate cake [40]. Regarding the trends of the filter loss curves, most curves have large slopes in the initial stage. However, after the initial stage, the slopes of the filter loss curves decrease. For the PTFE microporous membrane of pore diameter of 0.5 μm, the initial slope of mineral oil with 0.1 wt% PSL rubber nanogels is 27.53, and the second slope decreases to 2.93. The decreasing slope indicates that the permeability of filter cake decreases, which reflected that the compactness of the filtrate is enhanced. It also can be found that the second slopes of mineral oil with 0.1 wt%,0.5 wt%,1.0 wt% and 3.0 wt% PSL rubber nanogels were 2.93, 0.94, 0.91 and even 0.33, respectively. The slope decreased by approximately one order of magnitude, which reflected that the compactness of the filtrate enhanced markedly as the concentration of PSL rubber nanogels increased. Generally, the filter cake of drilling fluid needs to be thin, dense and flexible. As shown in Figure S1, the thickness of filter cake becomes thicker with the increase in PSL dosage. After adding 1 wt% PSL, the filter cake thickness increased to 1.18 mm. Generally, the greater the thickness of the filter cake indicated that more solid particles are trapped on the surface of filter paper to form a plugging layer. In addition, the pore diameter of the filter cake after the filtration test is tested, and the results are shown in Figure 3c. The pore diameter of original PTFE microporous membrane is 1113.7 nm. However, the pore diameter of filtration cake were significantly reduced after adding PSL rubber nanogels. The pore diameter of the filtration cake with 0.1 wt%, 0.5 wt% and 1.0 wt% PSL rubber nanogels were 989.5 nm, 799.4 nm and 462.9 nm, respectively. The same trend can be observed from other membrane with various pore diameters. As shown in the scanning electron microscopy of Figure 3c, the original polytetrafluoroethylene surface had many fine pores. When 0.1% PSL rubber nanogels was added, there were still a small amount of pores on the mud cake. However, the pores were basically invisible on the surface of the filter cake when 1% PSL rubber nanogels was added. The SEM images are consistent with the results of pore diameter analysis. In addition, it also can be seen that PSL rubber nanogels particles were becoming increasingly dense on the filtrate cake with the increase in the concentration of PSL rubber nanogels.

**Figure 6:**
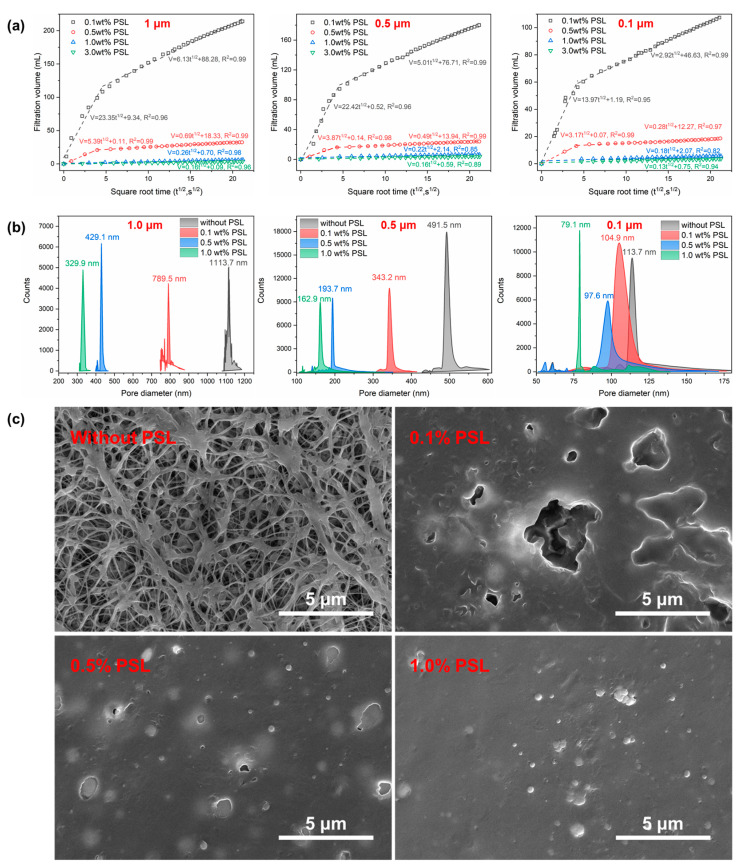
(**a**)The static filtration volume of W/O emulsion with different concentrations of PSL rubber nanogels as a function of square root of time, (**b**) the pore diameter distribution of the different polytetrafluoroethylene membrane after the filtration test, (**c**) SEM images of the polytetrafluoroethylene membrane (1.0 μm) after the filtration test. The magnification is 13,000 times.

The PSL rubber nanogels were dispersed into the inverse emulsion for the same evaluation, and the results are shown in Figure 6. The inverse emulsion group showed similar results to the mineral oil group. The slope of the filtration curve decreases with the prolongation of the experimental time and the increase in the concentration of nanogels, and the pore diameter of the filter cake decreases to become more compact with the increase in the concentration of nanogels. Slightly different is that the inverse emulsion with PSL rubber nanogels had lower filtration and a smaller pore diameter, which indicated that the plugging ability can be further improved by adding PSL into the emulsion. The results demonstrated that adding the PSL to mineral oil and inverse emulsion can improve the plugging effect.

### 2.6. Applied in Oil-Based Drilling Fluid

#### 2.6.1. Foaming Tests

The effects of PSL rubber nanogels on the foaming rate of mineral oil are shown in Table 1. In addition, the effect of SDS as a conventional emulsifier in emulsion polymerization was tested for comparison. The concentration of PSL is 1 wt% (solid content), while that of SDS is 0.01 wt% equal to the amount of reactive emulsifier in PSL. The PSL rubber nanogels prepared through soap-free emulsion polymerization showed much lower foamability than SDS. It should be noted that a low foaming tendency of additives on drilling fluid is required before their high functional properties. Therefore, the PSL n 56 without emulsifiers during preparation possesses the advantages of low foaming properties, meeting the drilling requirement.

#### 2.6.2. Drilling Fluid Properties

The rheological and filtration properties of ODF with different nanoparticles before and after aging at 180 °C are shown in Table 2. The concentration of different nanoparticles is fixed at 2 wt%. Compared with the original ODF, the AV, PV increased before and after aging test owing to the introduction of solid particles, which increased flow frictions between solid–solid, solid–liquid, and liquid–liquid layers. The YP dominates the hole cleaning efficiency and should be kept at a high value to carry drilling cuttings during circulation. As for the filtration loss, the FL_API_ and FL_HTHP_ of original ODF after aging were 3.6 mL and 10.2 mL, respectively, confirming that the original ODF had good filtration performance. On this basis, the experimental results showed that the FL_API_ and FL_HTHP_ of ODF decreased remarkably. The FL_API_ was reduced to 2.8 mL, 3.6 mL and 4.2 mL, and the FL_HTHP_ was reduced to 7.8 mL, 9.8 mL and 9.2 mL for the PSL, NS and PS, respectively. Therefore, it could be believed that the PS and NS had good effect on the filtration property of ODF and that the PSL attained a more effective filtration control capacity. Collectively, the PSL nanospheres are compatible with ODF without bad effects on drilling fluid properties and can even improve its rheological and filtration properties, especially after thermal aging, showing great potential for ODF.

## 3. Conclusions

In this work, PSL rubber nanogels were synthesized via soap-free emulsion polymerization as a plugging agent in ODF. The plugging performance and mechanism of PSL were investigated and compared with NS and PS nanospheres. The D_50_ of PSL is 541 nm; the initial thermal decomposition temperature is 356 °C, and the glass transition temperature (T_g_) is 102 °C. The nanoparticles dispersed in mineral oil are dynamically stable without large-scale settlement, even after storage for a week. The swelling behavior of nanoparticles in mineral oil can be controlled by altering the temperature and time. After adding the PSL, the emulsion stability at high temperature of W/O emulsion was improved because the nanogels can form a dense granular film at the oil–water interface to prevent droplets coalescing. Furthermore, the PSL can reduce filtration loss in mineral oil, inverse emulsions and ODF and has a strong adaptability to pores of different sizes. Moreover, the PSL without emulsifier are compatible with ODF and do not cause foaming. The PSL rubber nanogels in this work can be the candidate for the optimization of the plugging property of ODFs and have great potential in enhancing wellbore stability.

## 4. Materials and Methods

### 4.1. Materials

Styrene (St, 99.5 wt%) and lauryl acrylate (LA, 99 wt%) were purchased from the Aladdin Industrial Co., Ltd., China. Potassium persulfate (KPS, 99 wt%), sodium bicarbonate (NaHCO_3_, 99 wt%), calcium chloride (CaCl_2_, 99 wt%), N, N-methylene bisacrylamide (MBA, 99 wt%) and Span 80 were purchased from Sinopharm Chemical Reagent Co., Ltd., China. Styrene was washed by 20 wt% sodium hydroxide solution and then washed to neutral with deionized water. Other chemicals were used without further purification. Deionized water was self-made in the laboratory. Mineral oil, organoclay, modified fatty acid, lecithin and oxidized asphalt were purchased from the CNPC Greatwall Drilling Company. Hydrophobic nano silica (NS, 99 wt %, ~40 nm, specific surface area is 380 m^2^/g) was purchased from Hubei Huifu Nanmaterials Co., Ltd. Polystyrene (PS) nanospheres were prepared in the laboratory by emulsion polymerization, with an average particle size of ~300 nm.

### 4.2. Methods

**Figure 7:**
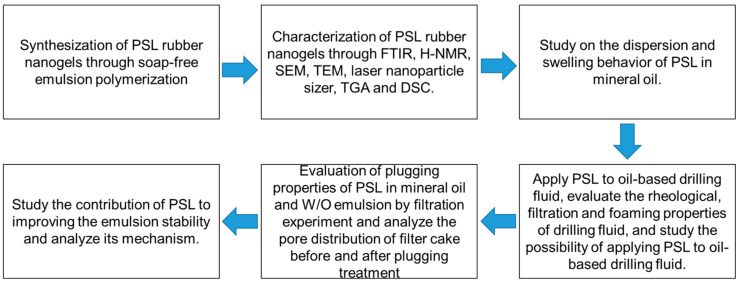
The workflow diagram of this research.

The following workflow diagram (Figure 7) presents the experimental steps for attaining the goal of this research.

### 4.3. Synthesis of PSL Rubber Nanogels

First, 0.065 g of NaHCO_3_ and 0.046 g of MBA was dissolved in 40 mL deionized water and transferred to a three-neck flask. The flask was immersed in water bath at 55 °C. Then a certain amount of St (8.33 g) and LA (2.4 g) were added into the solution dropwise under the protection of nitrogen, with a stirring speed of 2000 rpm. Finally, 0.081 g of KPS was added to initiate the polymerization reaction for 12 h. After reaction, the solution was centrifuged to separate the nanogels and washed with ethanol 3 times to remove impurities. The solid particles were freeze-dried to obtain the PSL rubber nanogels.

### 4.4. Characterization Methods of PSL

The PSL was dried under vacuum at 105 °C and then ground into powder. The FT-IR spectrum was measured at room temperature using a FTIR spectrometer (IRTracer-100-Shimadzu, Kyoto, Japan) and KBr pellets method. The frequency range was 400 cm−^1^ to 4000 cm^−1^, and the resolution was 4 cm^−1^. ^1^H NMR spectra of the PSL were recorded using a Brucker Advance DPX-300 spectrometer (Brucker, Germany) with a 30° pulse at 25 °C. Then, ~5 mg of samples was dissolved in 0.7 g of CDCl_3_. The thermal property (TG and DSC) of PSL was evaluated by the thermogravimetric analyzer (STA 409PC, Netzsch, Germany) under nitrogen flow with a temperature increase rate of 10 °C/min. The glass transition temperature was determined as the temperature at the midpoint of slope shifting. SEM (Hitachi S4800, Ibaraki, Japan) was used to observe the finer structure. Before observation, the samples were gold-coated using a sputter coater. A drop of diluted PSL solution was added on a carbon-supporting film. After drying, the sample was characterized by a TEM (Tecnai G2 F20, Hillsboro, OR, USA). The PSL nanogels were dispersed into mineral oil under ultrasound for 30 min, and then the particle size distribution was measured using a laser nanoparticle sizer (Zetasizer Nano Model ZS90, Malvern, UK).

### 4.5. Preparation of Fluid

#### 4.5.1. Reparation of Nonaqueous Suspension of PSL Rubber Nanogels

Nonaqueous suspensions, with mineral oil as the dispersion medium, were prepared by varying the PSL nanogels concentrations from 0.5 to 5.0 wt%. The suspensions were homogenized by ultrasonication to prepare the nonaqueous suspensions.

#### 4.5.2. Reparation of W/O Emulsions

W/O emulsions, with mineral oil as the continuous phase, deionized water as the dispersed phase and the Span 80 as the emulsifier, were prepared by varying PSL nanogels concentrations from 0 to 3.0 wt %. The volume ratio of mineral oil to deionized water was fixed at 8:2, and the emulsifier c oncentration was fixed at 2 wt% with respect to the total emulsion weight. The mixtures of mineral oil, deionized water, Span 80 and PSL nanospheres were homogenized by high-speed stirring at 10,000 rpm for 20 min to prepare the W/O emulsions.

#### 4.5.3. Preparation of Oil-Based Drilling Fluid

ODF was prepared according to the formula shown in Table 3. After adding each materials, the ODF was stirred at 10,000 r/min for 20 min. Then various nanoparticles (such as PSL nanospheres, PS, and NS) were added into ODF under stirring for 30 min to obtain nano-ODF, marked as ODF/PSL, ODF/PS, and ODF/NS, respectively.

### 4.6. Dispersion Stability Tests

The obtained nonaqueous suspensions of PSL rubber nanogels in Section 4.5.1 were left in a bottle to rest for 1 week at 30 °C, 80 °C and 130 °C to observe the settlement. In addition, the dispersion stability of suspension was also characterized based on the principle of dynamic light scattering (DLS) by TURBISCAN Lab Expert stability analyzer (Formulaction Company, Toulouse, France) at different temperatures, which is equipped with a pulsed near-infrared light source (λ = 880 nm) and synchronous optical detectors. The analysis of stability was carried out according to intensity variations of transmission light (ΔT) and backscattered light (ΔBS), which reflect the sedimentation and growth of polymer nanospheres in the suspension. The dispersion state of the PSL was investigated with an optical microscope.

### 4.7. Swelling Behavior of PSL Nanogels in Mineral Oil

A Nano ZS laser particle size analyzer (Malvern, UK) was used to monitor the particle size distribution of the obtained nonaqueous suspensions in Section 4.5.1., which were left at different temperatures (30 °C, 80 °C, 130 °C) to rest in a bottle for 1 week.

### 4.8. Emulsion Stability Test

The obtained W/O emulsions in Section 4.5.2 were left at different temperatures (20 °C, 40 °C, 60 °C, 80 °C) to rest in a bottle for 1 week to observe the non-emulsified volume. In addition, the emulsion stability (ES) was determined by using electrical stability measurement (DWY-2, Qingdao Tongchun Oil Instrument Co., Ltd., Qingdao, China) with an electrode distance of 1.55 ± 0.04 mm.

The morphology and mean drop size of the emulsion were investigated by an optical microscope (Leica DM4 B, Germany). The drop size was analyzed by Image J software.

To validate the dispersion of PSL at the oil–water interface, confocal laser scanning microscope measurements (CLSM, Leica TCS SPE, Leica, Germany, equipped with a laser operating at a wavelength of 532 nm) was utilized. Nile Red-tagged PSL nanogels (obtained by repeated centrifugation and then ethanol-washing) were added to emulsions while swirling at 10,000 rpm. The fluorescence picture of the W/O emulsion droplets was then examined with a fluorescence microscope.

### 4.9. Plugging Performance Tests

In the experiments, a polytetrafluoroethylene (PTFE) microporous membrane with a diameter of 90 mm was used. The filter media with pores of different sizes were selected to study the plugging performance of fluids at different scales. The pore sizes of the microporous membranes used as filtration mediums were 0.1 μm, 0.5 μm and 1.0 μm. An API filtrate meter (SD3, Qingdao Tongchun Oil Instrument Co., Ltd., Qingdao, China) was used to measure the filtration loss with time of the two fluids (i.e nonaqueous suspension and W/O emulsions) with different PSL concentrations. The testing conditions of the filter loss experiment were 25 °C at 0.69 MPa. The filtration volumes (FL_API_) were recorded every 10 s for 450 s.

### 4.10. Compatibility with Oil Based Drilling Fluid

#### 4.10.1. Foaming Tests

It is necessary to study the foam performance of ODFs, which has a great impact on the density, viscosity and filtration performance of drilling fluid. To study the effect of PSL on the foaming rate, there are the following main steps: First, we dispersed a certain amount of PSL nanospheres into 300 mL of mineral oil at a high speed of 10,000 rpm for 20 min and then pumped it for 3 min in a vacuum state. Then, the solution was poured into the measuring cylinder, and the sample volume was collected; the sample volume was calculated according to the volume-increasing proportion of the original sample size. The foaming rate was calculated as the ratio of volume increment to the original sample volume.

#### 4.10.2. Drilling Fluid Properties

ODF with various nanoparticles was stirred at high speed for 20 min to obtain a homogeneous suspension system and aged at 180 °C for 16 h to determine the rheological and filtration properties.

A six-speed rotating viscometer (ZNN-D6; Qingdao Haitongda, Qingdao, China) was used to measure the viscosity of WDF. At different rotation speeds of 600 and 300 rpm, the viscometer’s reading value (θ_600_, θ_300_) was noted. Calculations of the apparent viscosity (AV), plastic viscosity (PV) and yield point (YP) were done using Equations (9)–(11).

(9) $$\mathrm{AV}=\theta_{600}/2\;\left(\mathrm{mPa}\cdot s\right)$$

(10) $$\mathrm{PV}=\theta_{600}-\theta_{300\;}\left(\mathrm{mPa}\cdot s\right)$$

(11) $$\mathrm{YP}=0.48\left(\theta_{300}-\mathrm{PV}\right)\;\left(\mathrm{Pa}\right)$$

The filtration performance was investigated on an API filtration apparatus, and the filtration volume (FL_API_) was collected under a pressure of 0.69 MPa for 30 min. A JM-2 high-temperature and high-pressure filtration instrument (Qingdao Tongchun Oil Instrument Co., Ltd., Qingdao, China) was used to measure the high-temperature and high-pressure filtration (FL_HTHP_) of ODF at 180 °C. The differential pressure was 3.5 MPa, and the filtration time was for 30 min.

## Figures and Tables

**Table 1:** Foaming rate of different PSL rubber nanogels in mineral oil.

Sample	Original Volume (mL)	Final Volume (mL)	Foaming Rate (%)
Without SDS	300	310	3.33
With 0.01 wt% SDS	300	440	46.67

**Table 2:** Rheological and filtration properties of ODF with different nanoparticles before and after aging at 180 °C for 16 h.

Type	Condition	AV/mPa·s	PV/mPa·s	YP/Pa	Gel/(Pa/Pa)	FL_API_/mL	FL_HTHP_/mL
Original OBDF	Before aging	32	28	4	4/13	3.2	----
	After aging	39	34	5	5/14	3.6	10.2
With 3% PSL	Before aging	39	34	5	5/15	2.4	----
	After aging	41	36	5	5/15	2.8	7.8
With 3% NS	Before aging	48	42	6	6/17	2.8	----
	After aging	45	40	5	5/16	3.6	9.8
With 3% PS	Before aging	38	34	4	4/11	2.4	----
	After aging	41	37	4	3/8	4.2	9.2

**Table 3:** Formulation of oil-based drilling fluid.

Adding Order	Component	Function	Amount
1	Mineral oil	Continuous phase	240 mL
2	Modified fatty acid	Primary emulsifier	9.0 g
3	Span 80	Secondary emulsifier	6.0 g
4	Organoclay	Adjust rheology	3.0 g
5	Lecithin	Wetting agent	5.4 g
6	Oxidized asphalt	Fluid loss reducer	6.0 g
7	Brine (20 wt% CaCl_2_ solution)	Dispersed phase	60 mL

## Data Availability

Not applicable.

## Supplementary Materials

The following are available online at https://www.mdpi.com/article/10.3390/gels9010023/s1, Figure S1: Digital images and thickness of filtration cakes of mineral oil (top) and W/O emulsion (bottom). The concentration of PSL from left to right is 0.1 wt%, 0.5 wt%, 1.0 wt%, 3.0 wt%.
