# Supplementary material for: Styrene-Lauryl Acrylate Rubber Nanogels as a Plugging Agent for Oil-Based Drilling Fluids with the Function of Improving Emulsion Stability
Source: Gels. 2022 Dec 28;9(1):23. doi: 10.3390/gels9010023 (PMC9858097; doi:10.3390/gels9010023)
Supplement: Supplementary file 1 [file gels-09-00023-s001.zip › gels-2126286-supplementary.pdf]

gels

Supplementary Materials

Styrene-Lauryl Acrylate Rubber Nanogels as a Plugging Agent for Oil-Based Drilling Fluids with the Function of Improving Emulsion Stability

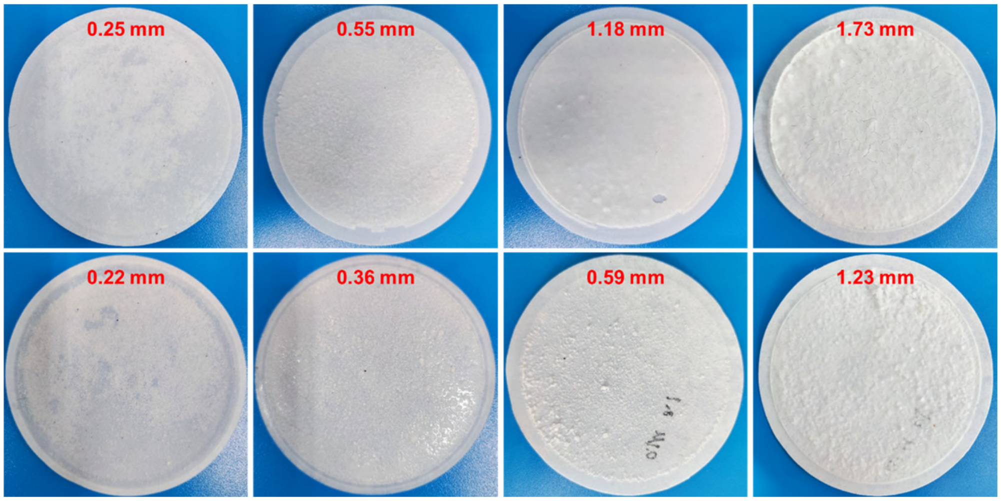

Figure S1. Digital images and thickness of filtration cakes of mineral oil (top) and W/O emulsion (bottom). The concentration of PSL from left to right is 0.1 wt%, 0.5 wt%, 1.0 wt%, 3.0 wt%.
